# Use of the international classification of diseases (ICD)-11 method applied to veterinary forensic pathology for coding the cause and manner of death in wildlife

**DOI:** 10.3389/fvets.2022.898721

**Published:** 2022-07-19

**Authors:** Cristina Marchetti, Anna Maria Cantoni, Luca Ferrari, Giovanni Maria Pisani, Attilio Corradi

**Affiliations:** Department of Veterinary Science, University of Parma, Parma, Italy

**Keywords:** ICD-11, cause, manner, death, veterinary forensic pathology, wildlife, “*The Link*”

## Abstract

The growth of human population has led, in recent years, to increasingly frequent contacts with the wild animals with which we share the territory, sometimes leading to negative interactions with them. The purpose of the study is to apply the codes contained in the 11th Revision of the International Classification of Diseases (ICD-11) method to investigate the cause and the manner of death, also to entrust the veterinarian with the task of recognizing and describing a suspected animal abuse as a sentinel indicator of violence toward humans and non-humans, thus expanding the concept of “One Health” from a forensic investigation perspective. The subjects recruited are wild mammals submitted for autopsy to the Pathology Unit of the Department of Veterinary Science, University of Parma, Italy, from 2015 to 2018. The manner and the cause of death of 167 wild animals of 16 different species have been investigated. When possible, an on-site inspection where the corpse was found was performed. Injuries were classified according to the on-line 11th Revision of the International Classification of Diseases method. Section 22 (Injury, poisoning or certain other consequences of external causes) was used to record the “immediate cause of death” (*cause of death*) and Section 23 (External causes of morbidity or mortality) was used to record the “underlying cause of death” (*manner of death*) for each animal. In most cases, death occurred as a result of road trauma but in some cases, abuse and voluntary killing were investigated. The recognition of non-accidental injuries is particularly important for both the defense in court of animals and for the connection between crimes committed against animals and against humans, known as “*The Link*”. The use of the ICD-11 method, as a sort of summary of the autopsy report, was confirmed to be of great value for the clarity and simplicity of processing the data collected also by veterinary pathologists. The veterinary pathologists can use this evidence-based method with the aim of creating a national register and therefore, to understand the real extent of the human impact on wildlife and document it in a scientific and statistically usable way.

## Introduction

In recent years there has been an increase in the presence of wild animals in areas inhabited by humans. This condition implies a new approach to nature that requires more attention in terms of environmental management, including various aspects of social life, such as garbage management (1), management of agricultural activities (2), and infrastructure planning. Besides all these, it is imperative to create deep knowledge based on the respect of all animals, both human and non-human.

Increasing human population has resulted in constant habitat fragmentation and isolation (3) and the subsequent road network proliferation, causing not only harmful and frequently lethal wildlife animals-vehicle collisions, but also exhibiting a different influence on wildlife, such as changes in behavior and even disappearance of local populations (4, 5).

Moreover, killing actions of individuals or entire animal and vegetal species that humans consider to be undesirable or even harmful, means that humans bring alteration of the ecosystem equilibrium.

When the Lisbon Treaty came into force in 2009, it amended the “Treaty on the Functioning of the European Union” (TFEU) and introduced the recognition of animals as sentient beings, meaning that they can feel pain and suffer, learn from experience, make choices, feel joy, and enjoy the company of others.

The empathy of European citizens and the growing culture of respect for animals, including wild ones, require an increase in the studies related to the causes of death of wildlife animals, and this is the aim of our investigation.

The absence of an individual medical history is the normal condition in which the forensic veterinary pathologist must operate during the ascertainment and attribution of the causes of death in wild animals. Intentional or unintentional killing of animals (manner of death) requires the forensic veterinarian pathologist to have specific skills for an appropriate investigation method. The work of the forensic veterinary pathologist must be scientific and independent to ensure the correct interpretation of the manner of death and the cause of death. The contribution of forensic veterinary pathology to the investigation and prosecution of wildlife animal crimes is to be considered a priority also in the awareness of the link between animal violence and violence against humans according to the well-known hypothesis of “*The Link*”. Forensic veterinary pathology is the application of knowledge of veterinary pathology to provide evidence (6) and is often an integral part of a wildlife crime investigation.

The cause of death is the specific injury or disease that leads to death. The WHO definition of “cause of death” is: “causal chain of events (disease or injury) that directly led to the death.” The “immediate cause of death” is represented by the “final event in the causal sequence that occurred closest to the time of death” while the “underlying cause of death” is the “initiating event in the causal sequence that occurred most remote from the time of death” which describes the circumstances surrounding the death and allows to define the manner of death. According to the WHO, the manner of death is the “classification of death based on circumstances surrounding it, i.e., suicide, homicide, accident, natural, or undetermined” (7).

The WHO definition of an “injury” is: “injuries are caused by acute exposure to physical agents such as mechanical energy, heat, electricity, chemicals, and ionizing radiation interacting with the body in amounts or at rates that exceed the threshold of human (and animal) tolerance.” Injury means physical or physiological bodily harm resulting from interaction of the body with energy (mechanical, thermal, electrical, chemical, or radiant or due to extreme pressure) in an amount, or at a rate of transfer, that exceeds physical or physiological tolerance. Injury can also result from the lack of vital elements, such as oxygen. Poisoning by and toxic effects of substances are included. The injury usually has rapid onset in response to a well-defined event (e.g., a car crash, hitting the ground after falling, ingesting a strongly alkaline liquid or an overdose of a medication). These events are often referred to as external causes of injury (7).

Data obtained from autopsy have been standardized and made manageable from a statistical point of view using the 11th Revision of the International Classification of Disease (ICD-11) (7).

In particular, the authors followed the classification contained in Section 22 “Injury, poisoning or certain other consequences of external causes” to record the main immediate cause of death for each subject and Section 23 “External causes of morbidity or mortality” to specify and codify the event that started the chain of events leading to death (underlying cause of death), on which the definition of the manner of death is hypothesized. In some circumstances (especially in the case of road trauma), the final cause of death is represented by several possible causes (e.g., polyvisceral and skeletal trauma). In these cases, complete multiple-causes coding is essential for a correct application of the ICD-11 method. The aim of this study is to investigate, using forensic scientific based evidence, the manner of death (resulting from knowledge of the underlying cause) and the cause of death in wildlife animals submitted for autopsy examination from 2015 to 2018 to the Pathology Unit of the Veterinary Teaching Hospital (VTH) of the Department of Veterinary Science, University of Parma, Italy. For some cases, judicial proceedings are still ongoing and therefore subject to confidentiality.

This is a proposal that the authors address to veterinary pathologists on the opportunity to use the codes validated by the World Health Organization (WHO) for the statistics of human mortality, also in veterinary forensic pathology. The forensic scientific based-evidence method, adopted in this study on wild animals, has the target of codifying the causes of death attributable to the anthropogenic impact on the ecosystem. The ICD-11 codes have proved to be applicable and consistent with the coding of the causes and manners of death of both wild and domestic animals in Sections 22 and 23, for the application of suspected causes of death of veterinary forensic pathology interest.

## Materials and methods

### Animals

The manner and cause of death of a total of 167 wild animals of 16 different species, listed in Table 1, were investigated.

**Table 1:** Mammal species chosen for the investigation and number of subjects.

**Mammal species**	**Nr. of subjects**
Badger (*Meles meles*, L, 1758)	17
Beech marten (*Martes foina*, E, 1777)	7
Coypu (*Myocastor coypus*, M, 1782)	2
Deer (*Cervus elaphus*, L, 1758)	2
Dormouse (*Glis glis*, L, 1766)	1
Fallow deer (*Dama dama*, L, 1758)	6
Fox (*Vulpes vulpes*, L, 1758)	6
Hare (*Lepus europaeus*, P, 1778)	8
Hedgehog (*Erinaceus europaeus*, L, 1758)	10
Mink (*Mustela vison*, S, 1777)	3
Mouflon (*Ovis aries musimon*, P, 1762)	3
Porcupine (*Hystrix cristata*, L, 1758)	6
Roe (*Capreolus capreolus*, L, 1758)	79
Squirrel (*Sciurus vulgaris*, L, 1758)	6
Weasel (*Mustela nivalis*, L, 1758)	2
Wolf (*Canis lupus*, L, 1758)	9

Most of the animals recruited are species of wildlife rescued in the Province of Parma, Emilia Romagna Region (Northern Italy) hospitalized at the Wildlife Rescue Center (WRC) Unit of the Veterinary Teaching Hospital (VTH), Department of Veterinary Science of University of Parma (Italy) from 2015 to 2018. Forty-seven animals were submitted dead, and forty-nine animals died spontaneously during hospitalization. Seventy-one animals were euthanized according to the American Veterinary Medical Association (AVMA) guidelines (2013) (8).

Euthanasia was chosen by VTH practitioners based on an ethical evaluation of wildlife animal physical conditions.

### Autopsy and laboratory analyses

Autopsy was performed in observation of internal standard operating procedures (6).

A segmental or total body radiographic study was performed before the autopsy in 26 cases of wildlife animals involved in traumatic injuries or victims of assault of firearms. Tissue samples for ancillary tests concerning epidemiological and sanitary surveillance were collected and referred to “Istituto Zooprofilattico Sperimentale della Lombardia e dell'Emilia Romagna” (IZSLER) for the specific protocol adopted by the Lombardy and Emilia-Romagna Regions (Italy) for the epidemiological surveillance of the territory:

- brain *in toto* for rabies virus detection (polymerase chain reaction, PCR) (carnivores);
- masseter and diaphragm muscles for *trichinella* spp. detection (digestion method governed by the Implementing Regulation (EU) 2015/1375) (carnivores);
- obex and medial retropharyngeal lymph-nodes for Chronic Wasting Disease (CWD) detection (enzyme-linked immunosorbent assay, ELISA) (ungulates).

The ancillary tests performed were:

- cytological (May-Grunwald-Giemsa) and histopathological routine forensic pathology investigation;
- specific histological test for lead residues search (sodium rhodizonate—NaR) (9). The detection of gunshot residues (GSR) and lead residues in histological tissue sections with the NaR staining technique is easy to perform. The NaR, a chelating agent, reacts with the lead forming a stable and colored complex. The method is highly sensitive so that low concentrations of lead in tissues can be easily demonstrated. The NaR reacts with heavy metal ions as lead, strontium, and barium present on the tissues around the wound, entry hole and into the intra-corporeal channel (9). The NaR test was applied in those cases in which the lesions were compatible with the suspicion of “assault by firearm” without bullet retention, therefore not detectable by radiological examination. The test was applied to both the suspected entry holes and the suspected exit holes of the bullet with the aim to provide additional information to distinguish between the two injuries and determine the direction of the shot;
- toxicological investigations: detection of anticoagulants requested to the IZSLER in 15 cases of carnivore species (wolf and fox);
- the diatom test, considered as the biological marker for the diagnosis of drowning in human forensic pathology (10). The diatom test protocol was performed according to the Italian guidelines for analysis of benthic diatoms for ecological status assessment of inland waters (11). In intravital conditions, the diatoms contained in the drowning medium can cross the alveolus capillary barrier, enter the pulmonary venous circulation and from there reach the left heart (12). When the cardiovascular system is active, the diatoms can reach other parts of the body, therefore, the presence of diatoms in target tissues such as lung, heart, liver, spleen, kidney, brain, and bone marrow (sternum), indicates that the victim was alive at the time of the dive. The diatom test provides useful information in order to distinguish drowning from post-mortem immersion. The detection of any aquatic microorganisms present both in the drowning medium and in the tissues of a drowning victim is sufficient to demonstrate the hematogenous dissemination of such particles from the lung by the heart pump during the violent process of asphyxiation. If a corpse is placed in water, the elements contained in the immersion fluid can passively enter the lungs and from there, passively cross the alveolar-capillary barrier during post-mortal processes but, in the absence of cardiac activity, they cannot reach the target organs (13). The method proved to be suitable for the identification of diatoms in the organs of the drowned animals supporting the final diagnosis of drowning (11, 14). The diatom test was conducted both on the target organs (lung, heart, liver, spleen, kidney, bone sternum medulla, and brain of a coypu and a mink), and on the drowning medium (10). The test can be considered positive when a minimum of 5 diatoms per 100 μL (a minimum of 20 diatoms is requested in lungs) is present in a sample (11).

In order to homogenize and standardize the data, the on-line ICD-11 method was employed. Section 22: “Injury, poisoning or certain other consequences of external causes,” was used to record the immediate cause of death for each animal and Section 23: “External causes of morbidity or mortality” was recorded to report the underlying cause of death, and consequently the definition of manner of death. When two or more conceivable causes of death are considered as multiple fatal injuries (e.g., polytrauma), more than one code was recorded (15). Euthanasia has never been cited as cause of death because euthanasia is a medical act, guided by the professional ethics of the veterinary surgeon, carried out in order to avoid unacceptable psycho-physical suffering and/or pain for the patient (16). The AVMA guidelines mention the conservation of wild animals and the impact of human activities on the fauna and environment and indicate veterinarians as the professionals entrusted with the task of caring for animals with reference to how to relieve pain and unnecessary suffering (17). Literature explains the difference between the cause of death (COD) and the reason for euthanasia (RFE) (18–21).

In some cases, an on-site investigation was conducted to collect information and samples from the corpse and from the environment, with the aim of not losing and valorizing information needful for drafting the final autopsy report.

## Results

In 39 cases (one coypu, two deer, six fallow deer, one fox, three minks, three mouflons, 14 roes, and nine wolves), on-site investigation sheets were applied. The data collected in a standardized way helped to understand the underlying causes that led to the death of the animal and allowed us to hypothesize the manner of death.

All ancillary tests performed for epidemiological surveillance protocols were negative (rabies, trichinosis and CWD). In two cases, a roe and a wolf, x-rays showed the presence of a radiopaque foreign body, identified as a bullet, localized in the *presternalis* region of the roe, and four radiopaque foreign bodies, at autoptic examination identified as hunting pellets, localized on the regions of the skull and neck of the wolf.

Based on the recent pathological history in the cases in which it was available (derived from on-site inspection or statements of witnesses), and the results of the autoptic examination, each subject was assigned one or more ICD-11 codes for the “immediate cause of death” and one or more codes for the “underlying cause of death.”

### Immediate causes of death

The immediate causes of death found and the relative ICD-11 codes are reported in Table 2. In some cases, multiple injuries were shown simultaneously or in rapid sequence after the traumatic event.

**Table 2:** Immediate causes of death and ICD-11 codes recorded in this study.

**Injury, poisoning, or certain other consequences of external causes**	**ICD-11 code**
Other specified infectious meningitis not elsewhere classified	1D01.Y
Myiasis	1G01
Sepsis with septic shock	1G41
Respiratory failure	CB41
Gastritis due to external causes	DA42.8
Diaphragmatic hernia	DD50.0
Hemorrhage, not elsewhere classified	MG27
Hypovolemic shock	MG40.1
Open wound of head, unspecified	NA01.Z
Fracture of vault of skull	NA02.0
Fracture of base of skull	NA02.1
Fracture of mandible, unspecified	NA02.7Z
Multiple fractures involving skull or facial bones	NA02.8
Diffuse injury of cerebrum	NA07.30
Crushing injury of head	NA08
Open wound of neck	NA21
Fracture of first cervical vertebra	NA22.0
Fracture of second cervical vertebra	NA22.1
Fracture of other specified cervical vertebra	NA22.2
Multiple fractures of cervical spine	NA22.3
Dislocation of cervical vertebra, unspecified	NA23.1Z
Open wound of thorax, unspecified	NA81.Z
Other specified fracture of rib, sternum or thoracic spine	NA82.Y
Injury of heart	NB31
Traumatic pneumothorax	NB32.0
Traumatic hemothorax	NB32.1
Certain injuries of lung	NB32.3
Multiple injuries of intrathoracic organs	NB32.7
Open wound of abdomen, lower back, or pelvis	NB51
Fracture of lumbar spine or pelvis	NB52
Fracture of lumbar vertebra	NB52.0
Fracture of the pelvic ring with incomplete disruption of posterior arch	NB52.2
Fracture of coccyx	NB52.11
Injury of spleen	NB91.0
Injury of liver	NB91.1
Injury of multiple intra-abdominal organs	NB91.B
Injury of urinary or pelvic organs	NB92
Multiple injuries of wrist or hand	NC5A
Crushing injury of shoulder or upper arm	NC17
Traumatic amputation at level between left elbow and wrist	NC38.4
Crushing injury of hip or thigh	NC77
Crushing injury of ankle or foot	ND18
Crushing injuries involving multiple body regions	ND34
Other injuries of leg, level unspecified	ND55
Traumatic shock, not elsewhere classified	NF0A.4
Effects of thirst	NF07.1


The immediate causes of death of the animals recruited in the present study are listed in Table 3.

**Table 3:** Distribution of the immediate causes of death and ICD-11 codes related to the animals recruited in the study.

**ICD-11**	**Badger**	**Beech marten**	**Coypu**	**Deer**	**Dormouse**	**Fallow deer**	**Fox**	**Hare**	**Hedgehog**	**Mink**	**Mouflon**	**Porcupine**	**Roe**	**Squirrel**	**Weasel**	**Wolf**
1D01.Y													2			
1G01													2			
1G41													1			
CB41			1				1			1						
DA42.8									3				1			
DD50.0													2			
MG27	1												2			
MG40.1							1									
NA01.Z										1			1			
NA02.0	1									1			4			2
NA02.1									3							1
NA02.7Z													5			
NA02.8		3			1				2				3	2	2	2
NA07.30	1	1	1												1	
NA08	3	1						1								
NA21	1															
NA22.0	1												3			
NA22.1	1							1			1		5			
NA22.2													1			
NA22.3														1		1
NA23.1Z				1												
NA81.Z									3		1					
NA82.Y	1	1		1		4					2	2	10	1		1
NB31	1					1					2		13			1
NB32.0						1	1				1	1	1			1
NB32.1	2	1				3	1	1			2	1	14	2		4
NB32.3													6			3
NB32.7	2							1	1				1			
NB51									4							
NB52	2					1	2			1		1	2			
NB52.0						1	1					1	4			
NB52.2	1						1						2			
NB52.11													1			
NB91.0													1			
NB91.1												2	13			2
NB91.B	1	1				3		2				1	15	1		3
NB92	2											1	4			
NC5A													1			
NC17													1			
NC38.4													2			
NC77	1					1		1				1	17			
ND18													3			
ND34	3							3					3	1		
ND55								1					4			
NF0A.4													3			
NF07.1													1	1		


Because few analyses of traffic accident injuries in animals are reported in the veterinary literature (22), we based our considerations on studies conducted in human forensics. Reading the injuries reported by the victims allowed us to reconstruct the phases of investment in human medicine translated to the anatomical characteristics of the animals (23–25). Depending on the size of the animal, the consequent lesions to the impact can be very different. Animals with a height at withers similar or superior to that of the bumper of the car or van that invests, suffer injuries consequent to the impact, projection and fall to the ground and sometimes to the sliding on the ground. In the impact phase, direct lesions produced by the impact against the front of the car are represented by extensive subcutaneous blood accumulation and muscle hematomas, fracture of skull or facial bones, column fractures and injuries of internal organs, mainly liver and heart. Fractures, mainly displaced and open, of the carpus, metacarpus, tarsus, metatarsus and phalanges, can be produced by projection and falling to the ground or against an object. This dynamic typically involves wolves, roes, young fallow deer, and young deer. The dynamic of an accident that occurred to an adult deer included an impact phase and a loading phase. The resulting lesions were dislocation of cervical spine and rib fractures. The dynamics of being run over by vehicles caused the death of 3 foxes and crushing lesions were evident on the pelvic region with extensive abrasions, fractures of the pelvic ring associated with multiple injury of the urinary tract, pelvic organs, intra-abdominal organs, and fractures of pelvic limbs. Animals with height at the wither inferior to that of the bumper can be subjected to injuries of being dragged on the ground and squashing by the passage of the wheels on the body that leads to crushing injuries. Porcupines, coypus, badgers, squirrels, and weasels are subject to this kind of dynamics. The lesions, in these cases, involve multiple regions of the body.

The immediate cause of death of the animals trapped in the fence barriers is traumatic shock and injury to the organs of the thoracic and abdominal cavities. Furthermore, large hemorrhagic spreads are observed in the subcutis and in the musculature.

In small mammals, a frequent cause of death is attack by cats whose bites cause perforating lesions in various regions of the body. Dogs are often responsible for attacks on small mammals, such as hedgehogs and hares, but also on larger animals such as roe deer in which the cause of death is represented by traumatic shock, extensive muscle lacerations, and hemorrhages.

Intraspecific assaults due to seasonal fights are frequent in cervids and mouflons. The causes of death recorded are head trauma and penetrating chest wounds causing pneumothorax, hemothorax, pulmonary laceration, hemopericardium and heart contusion. In two cases, death was consequent to a cerebral infection caused by wound complication and myiasis.

### Underlying causes of death

A total of 14 underlying causes of death, listed in Table 4, were recorded and all of them were linked to an interaction with humans and their activities. The number of animals dead as a consequence of road trauma is predominant (110 cases out of 167, namely 65.8%).

**Table 4:** Underlying causes of death, ICD-11 codes, and presumable manners of death recorded in this study.

**External causes of morbidity or mortality**	**ICD-11 code**	**Presumable manner of death**
Unintentional land transport road traffic injury event-public highway, street, or road	PA00 XE5NE	Unintentional
Unintentional land transport traffic event injuring a pedestrian-train	PA00 XE320	Unintentional
Unintentionally struck by moving object-mobile machinery or special purpose vehicle mainly used in agriculture	PA81 XE7X0	Unintentional
Unintentional striking against stationary object	PA82	Unintentional
Unintentionally caught, crushed, jammed, or pinched between objects	PA85	Unintentional
Unintentional drowning or submersion, while in body of water	PA90	Unintentional
Unintentional injury other than drowning following fall into body of water	PA92	Unintentional
Unintentional exposure to or harmful effects of substances chiefly non-medicinal as to source	PB36	Unintentional
Assault by being struck, kicked, or bumped by animal	PE11	Intentional
Assault by being bitten by animal	PE15	Intentional
Assault by projectile from firearm	PE20	Intentional
Assault by being struck by blunt object	PE40	Intentional
Assault by drowning or submersion, while in body of water	PE50	Intentional
Assault by exposure to or harmful effects of other or unspecified substances chiefly non-medicinal as to source	PE95	Intentional

Table 5 lists the causes of death and the related ICD-11 codes found in the animals examined.

**Table 5:** Distribution of the underlying causes of death defined by the ICD-11 codes and percentages of cases.

	**Badger**	**Beech marten**	**Coypu**	**Deer**	**Dormouse**	**Fallow deer**	**Fox**	**Hare**	**Hedgehog**	**Mink**	**Mouflon**	**Porcupine**	**Roe**	**Squirrel**	**Weasel**	**Wolf**	**Total**	**%**
ICD-11 code	17	7	2	2	1	6	6	8	10	3	3	6	79	6	2	9	167	100
PA00 XE5NE	17	7	1	1		1	3	6				6	56	3		9	110	65.8
PA00 XE320													2				2	1.2
PA81 XE7X0									1				3				4	2.4
PA82													3				3	1.8
PA85				1		2		1					3				7	4.2
PA90							1										1	0.6
PA92													1				1	0.6
PB36									3				1				4	2.4
PE15					1			1	6				4	3	2		17	10.2
PE11						3					3		5				11	6.6
PE20							1			1			1				3	1.8
PE40										1							1	0.6
PE50			1							1							2	1.2
PE95							1										1	0.6

Incidental findings were observed in two wolves who died due to road trauma: in the first animal, marks left by a lace around the neck were visible while in the second animal, ammunition was found in the muscle. Intentional entrapments are reported as the underlying cause of death in coypus.

**Figure 1:**
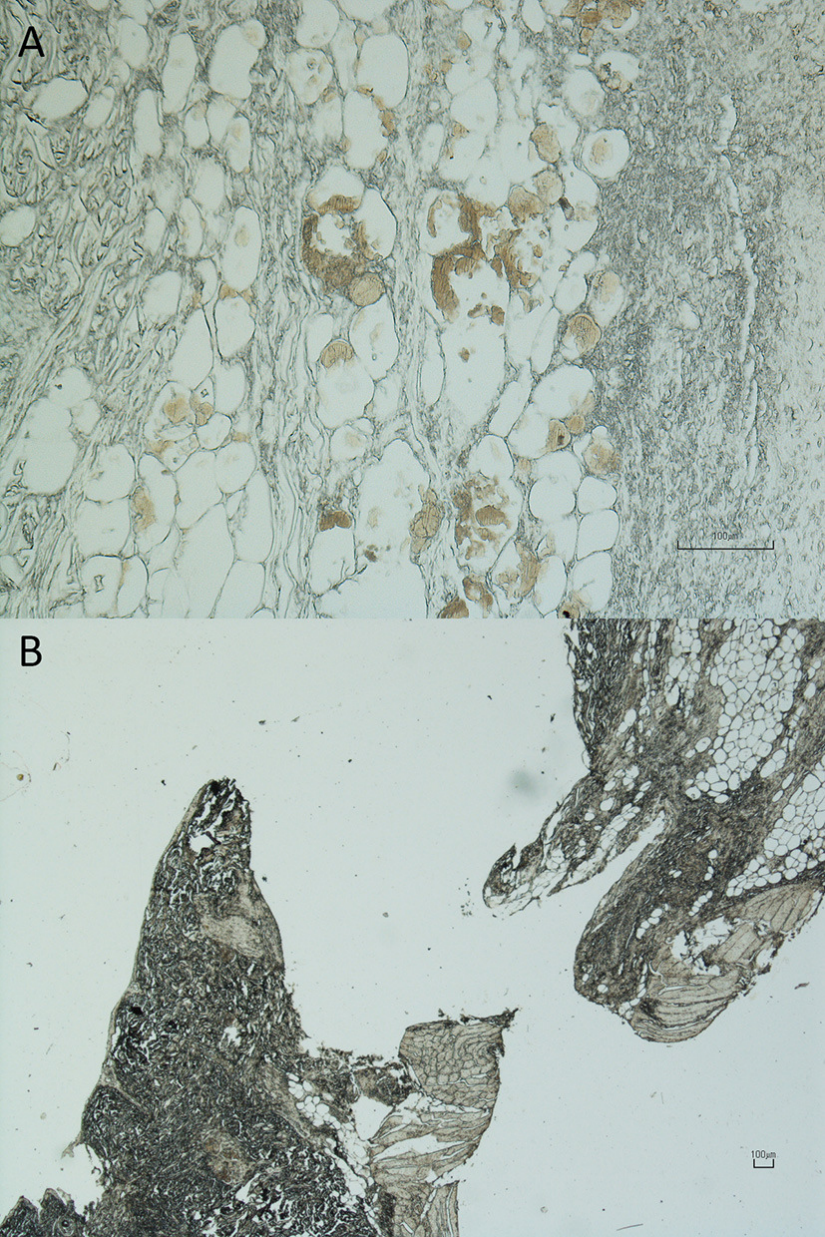
“Assault by projectile from firearm”: underlying cause of death—code PE20 of the International Classification of Diseases (ICD-11) method. Cutis and subcutis, fox. **(A)** Entry hole (10×), sodium rhodizonate (NaR) method: areas of brown color can be observed resulting from the bond between the dye and lead residues; **(B)** exit hole (2×), sodium rhodizonate (NaR) method: absence of binding areas between the dye and lead residues (Nikon Eclipse E800 microscope and Digital Sight DS-Fi1 Camera, Nikon Corp., Japan).

A female roe deer at the end of pregnancy was found abandoned in a field; she was hit by a single bullet which fractured both thoracic limbs at the carpal level. A fox was hit in the chest by lead bullets, hospitalized, and died due to lung wounds with pneumothorax and hemothorax. In both roe and fox, the bullets were not hold, and the diagnosis based on the characteristics of the lesions was confirmed by the sodium rhodizonate test that showed the presence of lead in the bullet entry hole and the absence of lead in the bullet exit hole as shown in Figure 1A (entry hole) and Figure 1B (exit hole). The histopathological results confirmed the nature of the lesions and the underlying cause of death “gunshot wound” was recorded.

**Figure 2:**
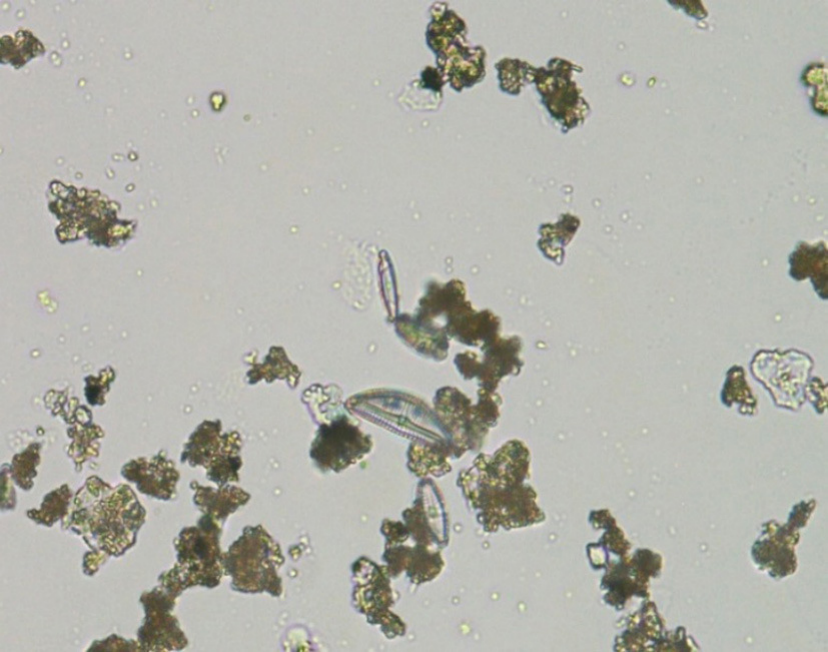
“Assault by drowning or submersion, while in body of water”: underlying cause of death — code PE50 of the International Classification of Diseases (ICD-11) method. Brain, *Myocastor coypus*. Digestive method (11). The image depicting a diatom of the family *Pennales* was obtained by an optical immersion microscope (100x, Nikon Eclipse E800, Nikon Corp., Japan) and captured by a Digital Sight DS-Fi1 Camera (Nikon Corp., Japan).

A coypu and a mink were found locked in a cage submerged in a ditch and in a lake, respectively. Evidence compatible with death by drowning was found at the dissection, then samples of target tissues were collected to be subjected to the diatom test. External exam of the coypu showed the presence of mud on the fur of all corporeal regions, abrasions and a wound on the hands and feet and avulsion of three nails of the hand. The autopsy showed the presence of mud on the oral and nasal cavity and in the trachea; rosy foam mixed with fragments of grass was present in the trachea and bronchi and water mix with grass was present in the stomach. The corpse of the mink gave off a sweetish odor, very similar to chocolate, then recognized as secretion of the perianal glands diluted in the drowning medium. The external exam showed the presence of mud on the ventral regions of the body. At the dissection we noted abundant presence of rosy foam into the trachea, bronchi and nasal cavity and abundant presence of water in the stomach. The elements collected at the place where the corpse was found and the evidence that emerged from the autopsy investigation are characteristic but not pathognomonic of drowning, therefore, other causes of death must be excluded in that lethal drowning is a complex event that requires the use of multiple sources of information, not just the data collected during autopsy. The diatom test is considered a fundamental aid in the diagnosis of drowning. The test performed both on the target organs, in particular lung, heart, liver, spleen, kidney, sternal medulla and brain of a coypu and a mink and in the drowning medium, contributed to the definition of the cause of death. The diatom test showed the presence of diatoms in target tissues of drowned victims (Figure 2); the minimum number of 30 diatoms was recovered in all cases investigated. In the case of a fox, a coypu and a mink, the samples of the drowning medium confirmed the presence of the same populations of diatoms found in the organs examined. As demonstrated by Piegari et al. the number and presence of diatoms in certain districts, such as the sternal medulla, can be considered as a valid tool in the definition of death by drowning (26). To confirm the diagnosis, the diatom test was applied to target tissues of a coypu and a mink, dead from causes other than drowning, with negative results (absence of diatoms). The test was performed in order to exclude diatoms contamination derived from the semi-aquatic habits but not ichthyophages (14) of the two species. The coypu and the mink corpses were conferred in rigor mortis whereas the fox was macerated but sufficiently preserved.

The presence of anticoagulants was found in two foxes. In one fox, this was the underlying cause of death whereas in the other fox, the toxic levels were sublethal because no signs of poisoning were observed during the autopsy and the underlying cause of death was road trauma. In both cases, the poison was identified as the anticoagulant brodifacoum.

## Discussion

Mortality data worldwide are coded according to the current International Classification of Diseases (ICD-11) method, published by the WHO. The system associates the cause of death with an alphanumeric code that corresponds to a particular disease or injury (7).

The ICD-11 is most useful for writing the veterinary forensic report to the same extent as it is for human victims (27). The proposal for forensic coding of injuries and causes of death on a traumatic basis stems from the experience in the field gained over the years in comparison with the consultation of publications about both veterinary and human forensic pathology. The ICD-11 tool presents a collection of events that cause injuries and is especially useful to categorize injuries according to whether or not they were deliberately inflicted and by who. The categories of the manner of death employable for a veterinary purpose are: unintentional (i.e., accidental), intentional (i.e., deliberate), and undetermined intent (7).

The category “interpersonal” (e.g., assault and homicide) can be translated into terms appropriate to veterinary criminology using the term “theriocide” coined by Beirne (28). Theriocide (or more generally, animal-*cide*) is the killing of an animal by a human. The field in which a theriocide act takes place derives from legislation, civilization, and cultural progress of each country. A theriocide may be due to an intentional or an unintentional purpose. It may involve active maltreatment or passive neglect. A theriocide act may be performed by a single individual, a group of people or an entire community and even be designed and perpetrated by the institutions. A theriocide, depending on the ethical and cultural heritage, can be recognized in the intensive farming type, in the hunting and fishing practice, in animal trafficking, animal experimentation, pollution, and human-induced climate change (28).

In some cases, determining whether the manner of death is intentional or accidental can prove particularly difficult for the forensic pathologist and the final answer can only be obtained from the final results of the investigations (29). In some other cases, the manner of death may remain undetermined although a cause of death is established (15). The role of the medical examiner is not to express an opinion but to document, interpret, and explain the results of the investigations carried out by the investigators and in court, in order to provide all the useful elements for the court to take a decision (6).

Generally, subjects hospitalized in a wildlife rescue center are victims of events that cause injuries, while the hospitalization of subjects suffering from pathologies is infrequent. This is mainly due to two reasons: (1) injured or distressed wild animals tend to hide and their condition is hardly reported to rescuers; (2) corpses are normally colonized by entomofauna or quickly consumed by scavengers. For these reasons, the first underlying cause of death of wild animals sent from a wildlife rescue center for the purpose of conducting post-mortem examinations, is road trauma. In the authors' casuistry, road trauma accounted for about 66% of causes of death in wild animals. Deaths were caused by a crash with car or vans whereas only two cases were victims of a railway accident. The death of wild ungulates did not reveal any behavioral contributing cause attributable to CWD infection. Data collected from the geographic area considered in this study certify the absence of an adequate system for the prevention of road accidents causing high risk for both animals and humans. The study of the height, type, and extent of the injuries makes it possible to conjecture the victim's initial position at the moment of impact and the direction of the forces exchanged (25). It is interesting to observe that 32% of roe body injuries were bilaterally localized in the ischial region and caudal region of pelvic limbs, which might suggest that the animal was already present on the road and therefore the driver could have avoided the animal. This consideration supports the fact that many accidents are caused by an inadequate speed related to visibility, specifically in night driving exactly as it happens in accidents involving motor vehicles and pedestrians (30) and underlines the high risk for both animals and humans. In terms of justice and public spending, it seems necessary to reconsider certain claims for compensation which should be considered as damage “to wildlife” and not “from wildlife.”

In the period of birth of roe deer and hares, which in our climate occurs in conjunction with the reaping, there are frequent cases of injuries caused by agricultural operating machines such as hay cutters and harvesters. Animals such as cervids and hares hide their newborn in the grass and remain with it only the time necessary for feeding. The wild youngsters, that for ethological reasons remain crouched and hidden in the long grass, results invisible to the drivers of the vehicles and can be subjected to severe or mortal injuries.

Three roes escaping from attacking dogs, impacted against iron or masonry barriers and suffered the dislocation of the cervical vertebrae. Barriers and fences are additional artificial elements installed along the pathway of the wildlife. The death due to imprisonment in these structures is slow and painful. The evidence collected during autopsy reveal the effect of an acute stress reaction, extended self-inflicted lesions made in attempts to gain freedom in combination with the protracted body dehydration. Plastic cables and wires can also cause death of the animal in attempting to free itself. A hare died under these circumstances. One fox fell into an unfenced reservoir where it drowned. This condition is obviously also a very serious danger for humans. One roe had access to a villa private park and fell into the swimming pool which had been emptied for the winter.

As solitary hunters, cats catch small prey like rabbits, weasels, small rodents, birds, and lizards. Cats completely dissociate their hunting behavior from their eating behavior. Cats show an extremely pronounced predatory instinct against moving objects. Even when properly fed, housecats allowed to go outdoor continue to catch and eat prey, although to a lesser degree than stray cats. Providing extra food does not eliminate the cat hunting behavior (31). Stray dogs or those left without a leash are a big danger to wildlife. Many wild animals are attacked and often suffer mortal injuries. Others fleeing can be hit by vehicles or as previously discussed, can impact against fixed obstacles. In our casuistry, dogs are responsible for assaults on roes and hedgehogs.

In our casuistry, the ICD-11 method proved to be applicable to poaching which is a frequent way of illegal wild animal killing. Specifically, the two wolves autopsied after a car accident showed evident signs of poaching as well as the female roe deer hit by a bullet and left agonizing in a field. In the case of the hit fox subjected to the NaR test, the wounding may have been due to legal hunting.

Over the years, coypus, an alien species, have been classified as invasive (invasive alien species, IAS) on the basis of the hypotheses that attributed to this species roles in the spread of diseases (e.g., *Leptospira* spp.), damage to agricultural activities and infrastructures and threat to biodiversity. The investigations conducted by the Istituto Zooprofilattico Sperimentale (IZS), as well as by University researchers, have excluded the role of the species in the spread of diseases (32, 33). Global impacts due to coypu excavations on earth structures are limited (4%) (34). Geologists are unanimous in attributing to the coypu lair only a secondary role in the instability of the embankments of the Po Valley (35). Regarding wetlands, the allegation of threat to plant and animal biodiversity, no evidence has been provided. Scientific literature invites to review and contextualize the concept of biodiversity itself (36). The law provides that the coypus are caught in trap cages and that these are regularly inspected to avoid unnecessary suffering to the animal which will then be killed in the manner permitted by law as soon as possible after capture. Despite the provisions of the law, there are reports of corpses in an advanced state of decomposition inside the cages. The current condition and the lack of control by the competent authorities have led many people to illegally kill trapped coypus with blunt objects or by raging on animals caught in a cage, piercing them with iron rods or simply letting them starve (32). In the case presented, as often happens, the animal was drowned by immersing the cage in the watercourse; attempts at self-liberation and agony were prolonged by the semi-aquatic nature of the species.

The three minks included in the study are part of a group of about one hundred subjects, released in circumstances still to be clarified, from an animal fur farm. Numerous have been victims of road traumatism. Those, subjected to autoptic exam, were killed by head trauma, by gunshot wound and drowning, configuring, also in this case, the crime of animal-cide.

The intentional diffusion of poisons in the environment is one of the major causes of death of wild and domestic animals. However, in case of wildlife, the finding of poisoned animals is mostly incidental because the victims tend to hide and thus the corpse is not easily found.

## Conclusions

The unintentional manners of death are linked to a rapidly expanding human footprint, comprised of anthropogenic land-use change and infrastructure, is profoundly affecting wildlife distributions worldwide.

Shackelford et al. recommend the use of the “cumulative-effects-assessment” (CEA) method to manage land-use changes because it offers a robust tool for informing on wildlife and habitat conservation (37). Road network proliferation causing habitat fragmentation, exhibits change in the behavior and disappearance of local populations and, relevant to this study, harmful and frequently lethal wildlife-vehicle collisions.

The recognition of the intentional manners of death (non-accidental injuries) is particularly important for both the defense in court of animals and for the well-known connection between crimes committed against animals and crimes committed against human, known as “*The Link*,” which proves that animal abuse links with other crimes. The World Organization for Animal Health (OiE) (38) and the World Small Animal Veterinary Association (WSAVA) (39) propose this topic to be considered as an aspect of the “One Health” concept, which should be applied to all animals, including wild animals. The reason why more space in this work is dedicated to the killing of a coypu through drowning is the alarming manifestation of violence spreading in recent years with particularly appalling episodes that requires a proper rethinking by the institutions and an invitation to accept the instances for scientific objectivity offered by several fronts.

The forensic site inspection played a role of primary importance in recognizing and understanding the events that led to death. The use of the ICD-11 method as a sort of summary of the autopsy report proved to be of great value for the clarity and simplicity of processing the data collected also by veterinary pathologists. The authors believe that the use of an on-site inspection and an autopsy is essential in order to accurately identify the cause of death, and they invite the veterinary surgeons to involve the forensic pathologists whenever possible. The authors invite the veterinary pathologists to use this evidence-based method with the aim of creating a national register and therefore, to understand the real extent of the human impact on the wildlife and document it in a scientific and statistically usable way.

## Data availability statement

The original contributions presented in the study are included in the article/supplementary material, further inquiries can be directed to the corresponding author.

## Ethics statement

Ethical review and approval was not required for the animal study because the enrolled animals were collected on the territory as corpses of spontaneously dead animals due to natural or accidental causes or animals that died spontaneously during hospitalization at the Veterinary Teaching Hospital (VTH) of the Department of Veterinary Science of University of Parma (Italy), or euthanized at the VTH according to the American Veterinary Medical Association (AVMA) guidelines for compassionate reasons.

## Author contributions

All authors listed have made a substantial, direct, and intellectual contribution to the work and approved it for publication.

## Funding

The present work received no specific funds. Institutional funds from the Department of Veterinary Science, University of Parma, Italy (CANTONI_MASTER_WILDLIFE_2020-2021) were received for the open access publication.

## Conflict of interest

The authors declare that the research was conducted in the absence of any commercial or financial relationships that could be construed as a potential conflict of interest.

## Publisher's note

All claims expressed in this article are solely those of the authors and do not necessarily represent those of their affiliated organizations, or those of the publisher, the editors and the reviewers. Any product that may be evaluated in this article, or claim that may be made by its manufacturer, is not guaranteed or endorsed by the publisher.
